# Identifying new safety risk of human serum albumin: a retrospective study of real-world data

**DOI:** 10.3389/fphar.2024.1319900

**Published:** 2024-01-15

**Authors:** Hui Lu, Yuwei Zhang, Pengcheng Liu

**Affiliations:** ^1^ Shanghai RAAS Blood Products Co., Ltd., Shanghai, China; ^2^ School of International Pharmaceutical Business, China Pharmaceutical University, Nanjing, China

**Keywords:** real-world data, human serum albumin, adverse reactions, safety, FAERS database

## Abstract

**Objective:** To mine and analyze the adverse reaction signals of human serum albumin (HSA) using the FDA adverse event reporting system (FAERS) database for the safe clinical use of this drug.

**Methods:** Data cleaning and analysis of adverse event reports in the FAERS database for a total of 76 quarters from Q1 2004 to Q4 2022 were performed using the reporting odds ratio (ROR), Medicines and Healthcare Products Regulatory Agency (MHRA), and Bayesian confidence propagation neural network (BCPNN). Gender-differentiated signal detection was used to investigate the gender differences in the occurrence of HSA adverse events.

**Results:** Through a combination of three methods, a total of 535 adverse event reports were identified. These reports involved 1,885 cases of adverse reactions, with respiratory, thoracic, and mediastinal disorders, as well as general disorders and administration site conditions, as the most common. One noteworthy new signal was the occurrence of transfusion-related acute lung injury. Additionally, gender-differentiated signals were present, with females experiencing paraesthesia, hypertension, pulmonary oedema, loss of consciousness, and vomiting.

**Conclusion:** This study has revealed that HSA poses a risk of causing transfusion-related acute lung injury. It has also been observed that adverse reactions, including paraesthesia, hypertension, pulmonary oedema, loss of consciousness, and vomiting, are more prevalent in females. These findings should be taken into account when using HSA in a clinical setting.

## 1 Introduction

Human serum albumin (HSA) is a biological product derived from plasma collected from hepatitis B vaccine-immunized healthy individuals by low-temperature ethanol protein isolation and heat-inactivated virus at 60°C for 10 h (Basu and Kulkarni, 2014) which can maintain plasma colloid osmolality, inhibit apoptosis and regulate trauma-induced inflammatory response, and has antioxidant activity (Chalidis et al., 2007). As an ideal natural colloid, HSA is now widely used clinically to increase blood volume, replenish plasma albumin, diagnose oedema or ascites of unknown etiology, aid in the treatment of cardiovascular diseases such as ischemic heart disease, heart failure, atrial fibrillation, stroke, etc. (Arques, 2018); chronic liver diseases such as renal insufficiency, decompensated cirrhosis, circulation in patients with ascites, and spontaneous bacterial peritonitis (Spinella et al., 2016; Jagdish et al., 2021); acute diseases such as hemorrhagic shock, burns, organ transplantation, therapeutic plasmapheresis; and chronic conditions of hypoalbuminemia such as malnutrition syndromes, nephrotic syndromes, and large-volume puncture procedures (Liumbruno et al., 2009).

Since its introduction in 1942, there has been significant interest in studying the safety of HSA due to its widespread use and high clinical dose as an injectable. Based on clinical experience over the past 35 years, it has been generally accepted that HSA is highly safe, to the point where its safety is rarely discussed (Tullis, 1977). This conclusion has been supported by large-scale pharmacovigilance studies (Haase et al., 2013; Tseng et al., 2020). Adverse reactions to HSA have been reported in the literature as isolated incidents (Facciorusso et al., 2011), with most clinical studies focusing on allergy-like reactions such as erythema, immediate hypersensitivity reactions, and anaphylaxis (Fujita et al., 2007; Wang et al., 2019; Daniel et al., 2020).

Over the past few years, the summary of product characteristics for HSA has been updated with additional information on adverse reactions. These reactions include cardiopulmonary issues such as dyspnea, arrhythmia, heart failure, blurred vision, and joint pain (U.S., 2023; FDA, 2023). Post-marketing safety studies on HSA have primarily been conducted between the 1990s and early 2000s (Vincent et al., 2003; Zhou et al., 2013). To better understand the safety of HSA, this study aims to analyze adverse event (AE) signals in the existing real-world big data and conduct a subgroup analysis based on gender. The results will provide a basis for the rational use of HSA in clinical practice, as well as warning revisions to the instructions.

## 2 Methods

### 2.1 Data sources and processing

This study was conducted using data from the FDA’s adverse event reporting system (FAERS), one of the largest adverse event databases in the world currently available to the public free of charge. It contains all mandatory and spontaneous reports of adverse events related to drug use since 2004 and provides open access to real-world raw data from the FDA on safety reports related to drugs, therapeutic biologics, and case-specific safety reports that can help researchers dig deeper into pharmacovigilance information (FDA., 2023).

The data for this study were obtained from the FAERS database, which has been publicly available since 2004 and is updated quarterly. Data from all ASCII data packages spanning 76 quarters, from Q1 2004 to Q4 2022, were extracted and imported into SAS 9.4 (Statistics Analysis System Institute Inc.) for data cleaning and analysis. According to the FDA’s recommended method for removing duplicate reports, reports with missing values for key information such as age and gender are excluded. After Q1 2019, a list of deleted reports exists in each quarterly packet, and after the data are de-weighted, reports are excluded based on the CASEID in the list of deleted reports.

After data cleaning and processing, we used “ALBUMIN HUMAN” to screen the PROD_AI and DRUG_NAME fields to obtain the adverse reaction reports of HSA as the “Primary suspicion” drug, and all other adverse reaction reports in the database were used as the reference group for categorical statistical analysis and signal detection analysis using SAS 9.4 for categorical statistical analysis and signal detection analysis.

Medical Dictionary for Regulatory Activity (MedDRA) has been used by drug regulatory authorities and the regulated pharmaceutical industry for the entry, retrieval, evaluation, and presentation of data throughout the regulatory process from pre-market to post-market application of human products. MedDRA ^®^ 26.0 was used in this study to standardize all preferred terms (PTs), which were then mapped to system organ classification (SOC) for further analysis.

### 2.2 Descriptive statistics

Descriptive statistics refers to the organization, overview, and calculation of a large amount of data information contained in a survey sample and is a method of summarizing and expressing quantitative data in a way that reveals the characteristics of the data distribution. This study categorized and statistically analyzed HSA-related adverse event reports for patient characteristics such as age, sex, and country.

### 2.3 Disproportionality analysis

The disproportionality analysis is a commonly used signal detection method based on the concept that a signal is considered to have been generated if a combination of drug-specific events in a database is significantly higher than the background frequency of the entire database and meets certain criteria. The calculation of the ratio imbalance measure is based on the construction of a 2 × 2 matrix table of behavioral target drugs and all other drugs, listed as target adverse events and all other adverse events, as shown in Table 1. Where A is the number of target adverse events for the target drug in the database, B is the number of other adverse events for the target drug in the database, C is the number of target adverse events for other drugs in the database, and D is the number of other adverse events for other drugs in the database.

**TABLE 1:** Two-by-two contingency table for Measure of Disproportion.

	Number of target AE reports	Number of other AE reports
Target drug	A	B
Other drugs	C	D

To avoid generating signals with high false positives, this study used the reporting odds ratio (ROR), medicines and healthcare products regulatory agency (MHRA), and Bayesian confidence propagation neural network (BCPNN) methods for signal detection (Evans et al., 2001; van Puijenbroek et al., 2002; Sakaeda et al., 2013). A positive signal is generated if all three methods yield positive results. If ROR, PRR, and IC-2SD signal values are larger, the signal is stronger. The specific algorithms and thresholds are shown in Supplementary Table S1.

After the disproportionality analysis, we compared the obtained signal results one by one with the description in the specification, and defined those that were not in the specification or whose descriptions were inconsistent as new signals.

### 2.4 Gender differential signal detection

In this study, gender differential signal detection (Tan, 2021) was used to investigate the presence of gender variability in the occurrence of adverse drug events. Its calculation is also based on the 2 × 2 matrix table, and the algorithm is based on the ROR. Where A is the number of target adverse events for the female patient group, B is the number of other adverse events for the female patient group, C is the number of target adverse events for the male patient group, and D is the number of other adverse events for the male patient group. The ratio of A to B (A/B) was divided by the ratio of C to D (C/D) to obtain the ROR, which was used to assess the relative risk of each drug-related adverse event by gender.

If A>5, C>5, A+C>50, and log_2_ROR>1, this indicates a higher risk of this side effect in females; if A>5, C>5, A+C>50, and log_2_ROR<-1, this indicates a higher risk of this side effect in males.

## 3 Results

### 3.1 Characteristics of the report

After data mining and pre-cleaning, a total of 535 HSA adverse event reports were reviewed in this study, involving 1,885 adverse events.

In terms of gender, there were more males (51.59%), in terms of age, the largest number of patients were between 65–120 years old (27.48%), followed by 27.10% of patients between 22–65 years old, in terms of regression, 128 patients (18.39%) progressed to hospitalization, and in terms of countries where they occurred, China (16.26%), United States of America (14.95%), Japan (11.03%) had more cases of adverse events (Table 2).

**TABLE 2:** Characteristics of AE reports associated with HSA.

Characteristics		N	Proportion (%)
Total		535	100.00
Gender	Male	276	51.59
	Female	247	46.17
	Unknown	12	2.25
Age	<1 month	2	0.37
	1 month–2 year	6	1.12
	2 years–12 years	13	2.43
	12 years–22 years	13	2.43
	22 years–65 years	145	27.10
	65 years–120 years	147	27.48
	Unknown	209	39.07
Outcome	Death	96	13.79
	Life-threatening	104	14.94
	Disability	10	1.44
	Hospitalization	128	18.39
	Congenital anomaly	2	0.29
	Other	307	44.11
	Required intervention	13	1.87
	Unknown	36	5.17
Occurrence country (n>10)	CN	87	16.26
	US	80	14.95
	JP	59	11.03
	CA	13	2.43
	GB	12	2.24

The 535 reports of adverse reactions to HSA collected in this study were mainly related to SOCs at the site of general disorders and administration site conditions, respiratory, thoracic and mediastinal disorders, skin and subcutaneous tissue disorders, and most commonly manifested as symptoms such as dyspnea, fever, chills, pruritus, hypotension, and pulmonary oedema. Details are provided in Supplementary Table S2.

### 3.2 Signal detection results

After analysis by the three methods, the signals generated were mainly related to 11 SOCs, which were ranked according to the number of instances of the signals involved, A, and shown in Table 3 for details.

**TABLE 3:** Overview of statistically significant IC-2SD, RORs, and PRRs (PT).

PT	A	IC-2SD	ROR (95%CI)	PRR	$\chi^{2}$
Respiratory, thoracic and mediastinal disorders (120)					
Dyspnoea	53	0.59	3.01 (2.29–3.95)	2.95	67.06
Pulmonary oedema	28	2.28	18.73 (12.89–27.20)	18.46	445.31
Hypoxia	17	1.51	15.29 (9.49–24.66)	15.17	211.01
Respiratory distress	12	0.91	13.18 (7.47–23.25)	13.10	122.28
Tachypnoea	10	0.95	24.62 (13.22–45.84)	24.49	202.34
General disorders and administration site conditions (113)					
Pyrexia	44	0.85	3.90 (2.89–5.26)	3.83	89.90
Chills	42	2.16	11.53 (8.49–15.56)	11.29	384.44
Chest discomfort	19	0.78	6.11 (3.89–9.61)	6.06	75.44
No therapeutic response	8	0.40	17.38 (8.68–34.82)	17.31	107.17
Skin and subcutaneous tissue disorders (89)					
Pruritus	35	0.66	3.74 (2.68–5.23)	3.69	66.41
Urticaria	33	1.48	7.44 (5.27–10.50)	7.33	174.40
Erythema	21	0.28	3.69 (2.40–5.67)	3.66	38.05
Vascular disorders (82)					
Hypotension	48	1.8	7.91 (5.94–10.53)	7.73	275.37
Flushing	18	0.64	5.65 (3.55–8.98)	5.60	63.62
Shock	8	0.16	11.08 (5.53–22.20)	11.04	63.35
Circulatory collapse	8	0.28	13.56 (6.77–27.17)	13.51	80.58
Immune system disorders (72)					
Anaphylactic reaction	29	2.41	20.82 (14.43–30.05)	20.52	519.14
Hypersensitivity	24	0.66	4.60 (3.07–6.87)	4.55	63.14
Anaphylactic shock	19	2.03	25.96 (16.52–40.80)	25.71	426.61
Investigations (72)					
Blood pressure decreased	37	2.47	16.97 (12.26–23.50)	16.66	529.31
Oxygen saturation decreased	21	1.65	13.23 (8.60–20.34)	13.09	222.67
Body temperature increased	8	0.16	11.08 (5.53–22.19)	11.04	63.34
hepatitis c antibody positivedecreased	6	0.25	548.4 (244.26–1231.22)	546.66	2695.45
Injury, poisoning and procedural complications (57)					
Infusion related reaction	23	1.75	13.12 (8.69–19.79)	12.97	242.37
Exposure during pregnancy	13	0.63	7.99 (4.63–13.79)	7.94	72.13
Foetal exposure during pregnancy	12	0.45	7.34 (4.16–12.94)	7.3	59.1
Transfusion-related acute lung injury	9	1.16	1109.90 (569.70–2162.31)	1104.61	8533.19
Infections and infestations (32)					
Sepsis	18	0.52	5.07 (3.19–8.06)	5.03	54.23
Hepatitis c	8	0.44	19.02 (9.49–38.09)	18.94	118.55
Suspected transmission of an infectious agent via product	6	0.24	220.98 (98.86–493.98)	220.28	1091.54
Cardiac disorders (28)					
Tachycardia	18	0.80	6.56 (4.12–10.44)	6.51	78.57
Cardio-respiratory arrest	10	0.20	7.33 (3.94–13.64)	7.29	48.22
Pregnancy, puerperium and perinatal conditions (17)					
Premature baby	11	0.86	14.85 (8.21–26.87)	14.77	127.78
Foetal death	6	0.11	46.49 (20.85–103.69)	46.35	222.47
Metabolism and nutrition disorders (14)					
Fluid overload	8	0.29	13.88 (6.93–27.80)	13.83	82.79
Hypervolaemia	6	0.20	95.65 (42.86–213.46)	95.35	468.30

Of all the PTs, with Transfusion-related acute lung injury (IC-2SD: 1.16; ROR: 1109.90; PRR: 1104.61; $\chi^{2}$ :8533.19), signal strength is the strongest and deserves attention (Table 3).

In SOC of respiratory, thoracic and mediastinal disorders, the strongest signals were Tachypnoea (IC-2SD: 0.95; ROR: 24.62; PRR: 24.49; $\chi^{2}$ : 202.34). Signals such as Pulmonary oedema (IC-2SD: 2.28; ROR: 18.73; PRR: 18.46; $\chi^{2}$ : 445.31), and Hypoxia (IC-2SD: 1.51; ROR: 15.29; PRR: 15.17; $\chi^{2}$ : 211.01) also warranted attention (Table 3).

In SOC of general disorders and administration site conditions, the strongest signals were chills (IC-2SD: 2.16; ROR: 11.53; PRR: 11.29; $\chi^{2}$ : 384.44). In SOC of skin and subcutaneous tissue disorders, the strongest signals were urticaria (IC-2SD: 1.48; ROR: 7.44; PRR: 7.33; $\chi^{2}$ : 174.40) (Table 3).

### 3.3 Gender differential signal analysis

To assess whether there is a gender difference in the occurrence of adverse events of HSA, the corresponding ROR, and log_2_ROR values were calculated for males and females, respectively, in the present study. The results showed that males were more likely to experience drug ineffective (ROR: 0.43; log_2_ROR: −1.22), whereas females experienced paraesthesia (ROR: 6.74; log_2_ROR: 2.75), hypertension (ROR: 2.53; log_2_ROR: 1.34), pulmonary oedema (ROR: 2.13; log_2_ROR: 1.09), loss of consciousness (ROR: 6.74; log_2_ROR: 2.75), vomiting (ROR: 2.24; log_2_ROR: 1.17), and maternal exposure during pregnancy (ROR: 11.28; log_2_ROR: 3.50), putting them at a higher risk, as shown in Table 4.

**TABLE 4:** Disproportionality analysis of signals stratified by case gender.

PT	SOC	A	ROR	log_2_ROR
Male				
Drug ineffective	General disorders and administration site conditions	7	0.43	−1.22
Female				
Pulmonary oedema	Respiratory, thoracic and mediastinal disorders	17	2.13	1.09
Maternal exposure during pregnancy	Injury, poisoning and procedural complications	10	11.28	3.50
Hypertension	Vascular disorders	9	2.53	1.34
Vomiting	Gastrointestinal disorders	8	2.24	1.17
Loss of consciousness	Nervous system disorders	6	6.74	2.75
Paraesthesia	Nervous system disorders	6	6.74	2.75

## 4 Discussion

In this study, we evaluated the post-marketing safety of HSA by disproportionality analysis and gender differential signal detection of the FAERS database. The results of the disproportionality analysis showed that the most common adverse events associated with HSA were dyspnoea, pyrexia, chills, hypotension, and pruritus, and transfusion-related acute lung injury was identified as a potential novel signal. Gender differential signal detection further revealed females are more likely to experience adverse events after HSA including paraesthesia, hypertension, pulmonary oedema, loss of consciousness, vomiting, and maternal exposure during pregnancy. Maternal exposure during pregnancy was not included in the discussion because it is a female-specific adverse reaction.

### 4.1 Potential new signals- transfusion-related acute lung injury

The results of this study found that Transfusion-related acute lung injury (TRALI) had the strongest signal strength. TRALI is a clinical syndrome with clinical features including acute dyspnea, hypoxemia, fever, hypotension, tachycardia, and leukopenia (Yu and Lian, 2023)]. One of the major causes of transfusion-related death is the occurrence of hypoxia-associated acute noncardiogenic pulmonary oedema. This can happen during or after a blood transfusion and is caused by damage to the pulmonary vasculature. Antibodies to human neutrophil antigens (HNAs) or human leukocyte antigens (HLAs) in the donor’s blood can bind to the recipient’s antigens and mediate this damage (Yu and Lian, 2023).

TRALI is a condition that is commonly associated with blood products, especially those that have a high plasma content such as plasma and platelets (van Stein et al., 2010). In addition, studies have found that plasma from female donors has a higher incidence of TRALI due to the presence of HLA antibodies in parturient donors (Toy et al., 2012). A study conducted by Sa K et al. discovered that age and female gender are factors associated with TRALI. Specifically, females who receive transfusions are more likely to develop TRALI than males (Kuldanek et al., 2019). It has been reported that females who give birth often have HLA I, HLA II, and granulocyte antibodies. When these antibodies come into contact with homologous antigens in transfused plasma, they trigger neutrophil activation and the release of oxidizing substances that can damage the lung endothelium (Popovsky and Moore, 1985; Kopko et al., 2001). This suggests that previous pregnancy experience plays a significant role in contributing to TRALI.

Our research indicates that HSA is strongly linked to TRALI, and that there is a higher likelihood of occurrence in females. Notably, all nine cases of TRALI in our retrospective analysis involved female patients. This was due to the absence of male patients, resulting in a sex-specific signal detection that could not be accurately calculated to generate a corresponding signal value. The manual emphasizes the cardiovascular overload caused by the use of HSA without mentioning TRALI, which requires careful identification. Further details can be found in Supplementary Table S3.

It is important to thoroughly understand a patient’s medical history, pregnancy, and health status before administering HSA. If a patient experiences shortness of breath or other related symptoms after taking the drug, it could be a sign of TRALI. In such cases, blood transfusion should be stopped immediately and the patient’s vital signs closely monitored. It is worth noting that transfusion-related pulmonary complications are often under-reported due to under-diagnosis. Healthcare professionals can use the Uniform Standardized Model Reporting Form and Recommendations for Transfusion-Related Pulmonary Complications (van Wonderen et al., 2023) to improve the collection of data on pulmonary transfusion reactions. This will facilitate better hemovigilance and related research.

### 4.2 Gender-differentiated signals

#### 4.2.1 Paraesthesia

Paresthesia is a condition where patients feel discomfort in certain body parts without any external stimulation. This discomfort is often described as sensations like ants crawling, electric shocks, numbness, heat or cold, tingling, or pins and needles. It is usually caused by sensory pathway stimulation and is commonly seen in individuals with peripheral neuropathy, spinal cord lesions, and brain disorders (Boulware, 2003).

A study conducted by Jeffrey T. and his team involved a retrospective analysis of patients with myasthenia gravis who underwent intravenous plasma exchange with 5% human albumin solution from 2005 to 2010. The study found that some patients experienced minor complications such as paresthesia (Guptill et al., 2013). It has been reported that abnormal sensation is an important clinical feature of peripheral neuropathy, and its prevalence is higher in females (Savettieri et al., 1993; Baldereschi et al., 2007; Kandil et al., 2012; Kruja et al., 2012). This prevalence does not vary with age (Baldereschi et al., 2007; Kruja et al., 2012), according to various studies. A screening study by Sharon G. Bruce also found that females with peripheral neuropathy have a higher risk of developing abnormal sensations (Bruce and Young, 2008). The present study’s findings are consistent with these results, indicating that females are at a higher risk of developing paresthesia after HSA use. It is essential for female patients to immediately notify their healthcare provider and be closely monitored if such symptoms occur.

#### 4.2.2 Hypertension

Hypertension is a medical condition that occurs when there is an increase in arterial blood pressure in the body’s systemic circulation. This is characterized by elevated systolic and/or diastolic pressure, with a minimum reading of 140 mmHg and 90 mmHg respectively. It can cause damage to vital organs like the heart, brain, and kidneys (Messerli et al., 2007).

Several studies have indicated that using HSA may lead to hypertension. In a prospective study conducted by C. Pusey (Pusey et al., 2010), data was collected on 154 patients who underwent plasma exchange with human albumin 4.5% solution (Zenalb 4.5). The study recorded possible treatment-related adverse effects, and all six of the patients experienced elevated blood pressure as an adverse effect.

Our study revealed that hypertension is not only associated with HSA, but also with gender, particularly in females. It is worth noting that although hypertension is more prevalent in adult females than in males in America, it becomes even more common in females after the age of 60, and this gap continues to widen with age. Similar studies conducted in Canada and other developing countries have also shown that hypertension is becoming more prevalent among older females (Prince et al., 2012; Robitaille et al., 2012).

During the perimenopausal period, the decrease in estrogen concentration can lead to increased blood pressure levels in hypertensive postmenopausal females. Sex hormones play a crucial role in regulating blood pressure, with both endogenous and exogenous estrogens known to lower it (Reckelhoff, 2001). The World Health Organization reports that most females enter menopause after the age of 45 (World Health Organization, 2023). As estrogen levels decrease in perimenopausal females, vasoconstrictors like endothelin and angiotensinogen are produced, while increased androgen levels alter the plasma ratio of circulating estrogens/androgens (Salpeter et al., 2006). This hormonal interaction leads to increased activation of the renin-angiotensin-aldosterone system, resulting in renal vasoconstriction, sodium reabsorption, and ultimately elevated blood pressure levels (Coylewright et al., 2008).

In our retrospective analysis, 14 patients developed hypertension. Out of these patients, nine were female, and six of them were older than 45 years (Supplementary Table S4). Therefore, middle-aged and elderly females should monitor their blood pressure closely after using HSA. They should pay attention to any increase in blood pressure and consult a doctor promptly.

#### 4.2.3 Pulmonary oedema

Pulmonary oedema is when there is an abnormal buildup of fluid in the lungs, which can lead to difficulty with breathing and respiratory failure (Murray, 2011). The use of HSA can sometimes cause pulmonary oedema, which has been warned against in instructions and highlighted in studies. A position statement on the use of HSA infusion for cirrhosis-related complications recommends monitoring cirrhotic patients closely for the development of pulmonary oedema (concordance score: 5; degree of consensus: very high) (Bai et al., 2023).

Differential signaling for gender in this study found that pulmonary oedema was associated with HSA use and occurred at higher risk in females. While transfusion-related acute lung injury may be the main cause of pulmonary oedema. This aligns with the research of Sa K et al., who also found that previous pregnancy and being female are potential risk factors for TRALI (Kuldanek et al., 2019).

HSA plays an important role in maintaining plasma colloid osmolality and should be administered slowly, as an infusion of high doses of HSA over a short period can lead to an excessive increase in circulating blood volume and an increased risk of pulmonary oedema (Jagdish et al., 2021). Kugelmas et al. (2023) suggest that attention must also be paid to the sodium content of HSA, as high sodium can lead to pulmonary oedema, and that one must be careful when combining it with vasoconstrictors. Special attention should be paid to the occurrence of lung injury and pulmonary oedema in female patients after the use of HSA.

The HSA is vital for maintaining a proper balance of fluids in the body, but it should be administered slowly to prevent any harmful effects. If given in high doses over a short period, it can lead to an excessive increase in blood volume and an increased risk of pulmonary oedema. It is important to note that the sodium content of HSA needs to be monitored, as high levels can cause pulmonary oedema (Kugelmas et al., 2023). Additionally, combining it with vasoconstrictors requires caution. Extra care should be taken when administering HSA to female patients, as there is a risk of lung injury and pulmonary oedema.

#### 4.2.4 Loss of consciousness

The term “loss of consciousness” is sometimes used to describe states, such as apoplectic seizures and complex partial seizures, in which people appear to be awake but unaware of themselves or their surroundings. Plum and Posner’s work on the content-related aspects of consciousness supports this usage. Various factors can cause partial or complete loss of consciousness, such as primary cerebral events, metabolic disorders, intoxication, psychogenic causes, or syncope (Plum and Posner, 1972; van Dijk et al., 2009; Sayk et al., 2019). Additionally, heart failure can also cause loss of consciousness, which can be life-threatening. This can be due to underlying ion channelopathies, cardiac inflammation, myocardial ischemia, congenital heart disease, cardiomyopathy, or pulmonary hypertension. Among non-cardiac causes, the most common one is vasovagal response (VVR), as noted by Juan Villafane (Villafane et al., 2021).

Research has studied various perspectives on risk factors for VVR. Studies have shown a higher risk of vasovagal syncope in certain blood donors, including first-time donors, young adults, and females (Trouern-Trend et al., 1999; Newman, 2002; Tomita et al., 2002; Bravo et al., 2011; Odajima et al., 2016). Takeshi Odajima conducted a gender subgroup analysis on factors contributing to VVR and found that females who donated to a single recipient had a higher risk of VVR. The gender difference may be related to the relationship between blood volume and extravascular space (Odajima et al., 2016). Additionally, lower BMI in females has also been reported as a risk factor for VVR, suggesting an association with interstitial space (Takanashi et al., 2012). Takanashi M (Tomita et al., 2002) suggested a higher risk of vasovagal syncope after a single blood donation in females during the blood donation process, and even a higher risk of VVR in females over 45 years of age (Farquhar et al., 2000).

Based on the results of the study, it seems that women are more likely to experience loss of consciousness after using HSA. This finding should be studied further. Our analysis of six cases of loss of consciousness (excluding one case with missing age information) showed that all the affected individuals were elderly women aged 60 years or older. Additional details regarding the cases can be found in Supplementary Table S5. Therefore, healthcare providers should pay close attention to elderly female patients who have used HSA. It is important to monitor patients’ vital signs carefully and take appropriate action promptly if syncope or other symptoms occur.

#### 4.2.5 Vomiting

Our findings indicate that females have a greater likelihood of experiencing vomiting as a result of HSA usage. Vomiting entails the forceful expulsion of stomach contents through the mouth, with chronic nausea and vomiting being persistent symptoms lasting for 4 weeks or more. Acute nausea and vomiting, on the other hand, are typically characterized as symptoms lasting for 7 days or less (Hasler and Chey, 2003).

Research conducted in the general population has revealed that females experience a higher prevalence of gastrointestinal symptoms compared to males (Lacy et al., 2018). A prospective study by Luciana Ferreira Silva (Silva et al., 2012) found that females undergoing continuous hemodialysis for MHD displayed a higher prevalence of nausea, vomiting, and decreased appetite than their male counterparts. It is possible that such differences in symptoms between genders are partially due to females being more likely to report feeling unwell, both physically and psychologically.

Gastroparesis is a medical condition that causes symptoms such as nausea, vomiting, and abdominal swelling. Research has shown that 70%–90% of patients with various types of gastroparesis (including diabetes mellitus, idiopathic, postoperative, and viral infections) are female (Jung et al., 2009; Parkman et al., 2011). The reason for this higher susceptibility in females is not clear, but it may have multiple causes. These include higher levels of sex steroid hormones, decreased expression of neuronal NO synthase, slower colonic transit time, altered serotonergic signaling, decreased sinus contractility, impaired uterine regulation and sensation, and increased visceral hypersensitivity (Gonzalez et al., 2020). These findings support the conclusion of the present study, which shows that females are more likely to experience vomiting after using HSA. Therefore, patients with gastrointestinal issues should use HSA with caution and monitor their health closely.

### 4.3 Limitations

It is important to note that the FAERS database is a self-reporting database, which means that it does not collect every single report of an adverse event or medication error related to a particular drug. This may result in incomplete data that does not accurately reflect the incidence of adverse events in all populations.

In addition, the database may not always include important indicators, such as gender, or have other reporting quality issues that could affect the reliability of the results. For example, we reviewed the TRALI reports in this study, which consisted of 9 cases, only 3 of which had a specific route of administration as intravenous drip and lacked data otherwise available for further analysis, which hampered our further analysis of the reports.

In addition, the signals of AEs identified by disproportionality analysis may only suggest a statistical association between the drugs and the AEs. Further research and evaluation are needed to establish their causality. This study analyzed only the reports of HSA use and did not take into account the dosage and combination of drugs. What’s more, most of the reported countries are from CN, US and JP, and there may be ethnic differences in the data.

## 5 Conclusion

This study used signal detection in the FAERS database to assess the safety of HSAs in real-world situations. The focus was on drug safety signals. The results showed that the majority of adverse reactions associated with HSA included chills, pruritus, hypotension, fever, dyspnea, and pulmonary oedema. Most of these were already known signals. However, transfusion-associated acute lung injury requires further attention as a potential new signal.

It is possible that women may experience more negative reactions to HSA, such as tingling sensations, high blood pressure, fluid in the lungs, loss of consciousness, and vomiting. This risk is higher for older women who may experience hypertension and loss of consciousness. These symptoms should not be taken lightly and require close monitoring during HSA treatment. If a patient experiences any of these serious side effects, it is essential to reduce the dosage or stop the treatment immediately. It is important to note that the study’s results are solely statistical associations and not a definitive cause-and-effect relationship. Further research is needed to confirm these findings.

## Data Availability

Publicly available datasets were analyzed in this study. This data can be found here: https://fis.fda.gov/extensions/FPD-QDE-FAERS/FPD-QDE-FAERS.html.
