# Supplementary material for: Identifying new safety risk of human serum albumin: a retrospective study of real-world data
Source: Front Pharmacol. 2024 Jan 15;15:1319900. doi: 10.3389/fphar.2024.1319900 (PMC10825956; doi:10.3389/fphar.2024.1319900)
Supplement: Supplementary file 1 [file DataSheet1.docx]

Identifying new safety risk of human serum albumin:

a retrospective study of real-world data

Hui Lu^1^, Yuwei Zhang^2^, Pengcheng Liu^2*^

*** Correspondence:** Pengcheng Liu, [liupcmail@163.com](mailto:liupcmail@163.com)

**Supplementary table 1 Measure of Disproportionality and signal generation criteria**

| **Method** | **Formula** | **Threshold** |
| --- | --- | --- |
| **ROR** | $ROR=AD/BC$  $ROR 95\%CI=e^{ln(ROR)\pm1.96\sqrt{(\frac{1}{A}+\frac{1}{B}+\frac{1}{C}+\frac{1}{D})}}$ | A≥3  $ROR$ *95%CI*>1 |
| **MHRA** | $PRR=\frac{A/{(A+B)}}{C/{(C+D)}}$  $PRR 95\%CI=e^{ln(PRR)\pm1.96\sqrt{\frac{1}{A}-\frac{1}{A+B}+\frac{1}{C}-\frac{1}{C+D}}}$  $\chi2=\frac{\left( \left\vert AD-BC \right\vert-N/2 \right)^{2}\times N}{(A+B)(C+D)(B+D)(A+C)}$ | A≥3  *PRR*≥2  $\chi2$≥4 |
| **BCPNN** | $IC={log}_{2}\frac{A(A+B+C+D)}{(A+B)(A+C)}$  $\gamma$*=*$\gamma_{ij}\frac{(N+\alpha)(N+\beta)}{\left( A+B+\alpha_{i} \right)\left( A+C+\beta_{j} \right)}$  $E(IC)={log}_{2}\frac{(A+\gamma_{ij})(N+\alpha)(N+\beta)}{(N+\gamma)(A+B+\alpha_{i})(A+C+\beta_{j})}$  $V(IC)=\frac{1}{{(log2)}^{2}}\{\left[ \frac{N-A+\gamma-\gamma_{ij}}{\left( A+\gamma_{ij} \right)\left( 1+N+\gamma\right)} \right]+\left[ \frac{N-A-B+\alpha-\alpha_{i}}{\left( A+B+\alpha_{i} \right)\left( 1+N+\alpha\right)} \right]+\left[ \frac{N-A-C+\beta-\beta_{j}}{\left( A+C+\beta_{i} \right)\left( 1+N+\beta\right)} \right]\}$  $SD=\sqrt{V(IC)}$  *IC-2SD*=*E*(*IC*)-2$SD$ | A≥3  *IC-2SD*＞0 |

Note: $\gamma$,$\gamma_{ij}$ are the Dirichlet distribution parameters; $\alpha_{i}$, $\alpha$,$\beta_{j}$, $\beta$ are Beta distribution parameters; *SD* is standard deviation; *IC-2SD* is the lower limit of *IC* 95%*CI*; hypothesis $\alpha=\beta=2$, $\gamma_{ij}=\beta_{j}=\alpha_{i}=1$.

**Supplementary table 2 Distribution of AEs in the reports (n≥10)**

| **SOC** | **PT** |
| --- | --- |
| General disorders and administration site conditions (261) | Pyrexia (44); Chills (42); Drug ineffective (25); Chest discomfort (19) |
| Respiratory, thoracic and mediastinal disorders (225) | Dyspnoea (53); Pulmonary oedema (28); Hypoxia (17); Respiratory distress (12); Tachypnoea (10); Respiratory failure (10) |
| Investigations (192) | Blood pressure decreased (37); Oxygen saturation decreased (21); Heart rate increased (13) |
| Skin and subcutaneous tissue disorders (179) | Pruritus (35); Urticaria (33); Rash (26); Erythema (21); Hyperhidrosis (13) |
| Injury, poisoning and procedural complications (152) | Infusion related reaction (23); Exposure during pregnancy (13); Foetal exposure during pregnancy (12); Off label use (12); Maternal exposure during pregnancy (11); Product use in unapproved indication (10) |
| Vascular disorders (122) | Hypotension (48); Flushing (18); Hypertension (13) |
| Nervous system disorders (109) | Headache (12) |
| Immune system disorders (99) | Anaphylactic reaction (29); Hypersensitivity (24); Anaphylactic shock (19) |
| Infections and infestations (96) | Sepsis (18) |
| Gastrointestinal disorders (94) | Nausea (20); Abdominal pain (14); Vomiting (12) |
| Cardiac disorders (93) | Tachycardia (18); Cardiac arrest (12); Cardio-respiratory arrest (10) |
| Metabolism and nutrition disorders (47) | / |
| Renal and urinary disorders (38) | Renal impairment (11); Renal failure (10) |
| Pregnancy, puerperium and perinatal conditions (33) | Premature baby (11) |
| Product issues (28) | / |
| Blood and lymphatic system disorders (27) | / |
| Musculoskeletal and connective tissue disorders (14) | / |

**Supplementary table 3** **Specific cases of Transfusion-related acute lung injury reported**

| **Case** | **Gender** | **Age** | **Adverse reactions** | **Indication** |
| --- | --- | --- | --- | --- |
| 1 | F | 84 | agitation, dyspnoea, flushing, hypertension, oxygen saturation decreased, paraesthesia, transfusion-related acute lung injury | plasmapheresis |
| 2 | F | 84 | agitation, body temperature increased, dyspnoea, flushing, hypertension, hypotension, oxygen saturation decreased, paraesthesia, pulmonary oedema, transfusion-related acute lung injury, unresponsive to stimuli | hyperviscosity syndrome, plasmapheresis |
| 3 | F | 4 | cardiac arrest, transfusion-related acute lung injury | mucopolysaccharidosis i |
| 4 | F | 52 | transfusion-related acute lung injury | plasmapheresis, kidney transplant rejection, antibiotic prophylaxis, prophylaxis against transplant rejection, antiviral prophylaxis |
| 5 | F | / | oxygen saturation decreased, respiration abnormal, transfusion-related acute lung injury, wheezing | ruptured ectopic pregnancy, haemorrhage, hypovolaemic shock, oxygen saturation decreased |
| 6 | F | 16 | cardiac arrest, chest pain, transfusion-related acute lung injury | myasthenia gravis |
| 7 | F | 16 | air embolism, cardiac arrest, circulatory collapse, disseminated intravascular coagulation, immune system disorder, pneumonia, pulmonary oedema, systemic inflammatory response syndrome, transfusion-related acute lung injury, tricuspid valve incompetence | myasthenia gravis |
| 8 | F | 56 | acute respiratory distress syndrome, cardiac failure congestive, fluid overload, pneumonia aspiration, pulmonary oedema, sepsis, transfusion-related acute lung injury | / |
| 9 | F | 56 | acute respiratory distress syndrome, cardiac failure congestive, cerebral infarction, dehydration, pneumonia aspiration, pulmonary oedema, sepsis, transfusion-related acute lung injury | subarachnoid haemorrhage |

*Abbreviatons: “F”, female.*

**Supplementary table 4** **Specific reports of female patients with hypertension**

| **Case** | **Age** | **Adverse reactions** | **Indication** | **dechallenge** | **rechallenge** |
| --- | --- | --- | --- | --- | --- |
| 1 | 84 | agitation, dyspnoea, flushing, hypertension, oxygen saturation decreased, paraesthesia, transfusion-related acute lung injury | plasmapheresis | / | / |
| 2 | 84 | agitation, body temperature increased, dyspnoea, flushing, hypertension, hypotension, oxygen saturation decreased, paraesthesia, pulmonary oedema, transfusion-related acute lung injury, unresponsive to stimuli | hyperviscosity syndrome, plasmapheresis | Y | U |
| 3 | 83 | atelectasis, blood pressure decreased, blood pressure fluctuation, chronic obstructive pulmonary disease, drug intolerance, hypertension, incorrect dose administered, infusion related reaction, post procedural complication, pyrexia, respiratory failure | colitis ischaemic, peritonitis | U | U |
| 4 | 69 | acute pulmonary oedema, hypertension, hypoxia, skin discolouration | t-cell lymphoma stage iii | U | U |
| 5 | 68 | bacteraemia, chills, cold sweat, hyperhidrosis, hypertension, hyperthermia, medication error, suspected transmission of an infectious agent via product | plasmapheresis | Y | N |
| 6 | 62 | headache, hypertension, hyperthermia, malaise, renal pain, septic shock, tachycardia, tremor | hypoproteinaemia, hypertension, swelling, cardiac failure congestive, abdominal pain upper, fluid retention | U | U |
| 7 | 42 | abdominal pain, blood pressure diastolic decreased, diarrhoea, drug interaction, hypertension, nausea, procedural complication, vomiting | plasmapheresis | N | Y |
| 8 | 41 | bradycardia, hyperhidrosis, hypertension | plasmapheresis | U | U |
| 9 | 19 | abdominal pain, blood pressure diastolic decreased, diarrhoea, drug interaction, hypertension, nausea, vomiting | plasmapheresis | N | Y |

*Abbreviatons: “Y”, yes;“ N”，no; “U”， Unknow, null value or does not apply.*

**Supplementary table 5** **Specific situations in which loss of consciousness has been reported**

| **Case** | **Gender** | **Age** | **Adverse reactions** | **Indication** |
| --- | --- | --- | --- | --- |
| 1 | F | 80 | brain natriuretic peptide increased, c-reactive protein increased, hyperhidrosis, hyperinsulinaemia, hypoglycaemia, insulin autoimmune syndrome, loss of consciousness, palpitations, renal impairment | blood pressure decreased |
| 2 | F | 69 | blood pressure decreased, chills, feeling abnormal, loss of consciousness, nausea, pyrexia | plasmapheresis, pemphigoid |
| 3 | F | 68 | anaphylactic shock, foaming at mouth, hypoxia, loss of consciousness, pulmonary embolism | cardiac failure, generalised oedema, hepatic cirrhosis |
| 4 | F | 64 | dizziness, hypersensitivity, incontinence, loss of consciousness, nystagmus, vomiting | ascites, hepatic cirrhosis, hepatic function abnormal |
| 5 | F | 64 | anaphylactoid reaction, dizziness, foaming at mouth, loss of consciousness, urticaria | hepatic cirrhosis, ascites |
| 6 | F | / | cardiac arrest, cardiac failure, drug ineffective, loss of consciousness, multiple organ dysfunction syndrome, overdose, pulmonary oedema, respiratory arrest, transfusion-related circulatory overload, volume blood increased | hypoproteinaemia |

*Abbreviatons: “F”, female.*
